# Investigating causal effects of pupil size on visual discrimination and visually evoked potentials in an optotype discrimination task

**DOI:** 10.3389/fnins.2024.1412527

**Published:** 2024-10-01

**Authors:** Hsin-Hua Chin, Ying-Hsuan Tai, Rachel Yep, Yi-Hsuan Chang, Chun-Hsien Hsu, Chin-An Wang

**Affiliations:** ^1^Eye-Tracking Laboratory, Shuang Ho Hospital, Taipei Medical University, New Taipei, Taiwan; ^2^Department of Psychology, Chung Yuan Christian University, Taoyuan, Taiwan; ^3^Department of Anesthesiology, School of Medicine, College of Medicine, Taipei Medical University, Taipei, Taiwan; ^4^Department of Anesthesiology, Shuang Ho Hospital, Taipei Medical University, New Taipei, Taiwan; ^5^Sunnybrook Research Institute, University of Toronto, Toronto, ON, Canada; ^6^Institute of Cognitive Neuroscience, National Central University, Taoyuan, Taiwan

**Keywords:** pupillometry, visual discrimination, visual sensitivity, visuocortical activity, pupil size

## Abstract

Pupil size primarily changes to regulate the amount of light entering the retina, optimizing the balance between visual acuity and sensitivity for effective visual processing. However, research directly examining the relationship between pupil size and visual processing has been limited. While a few studies have recorded pupil size and EEG signals to investigate the role of pupil size in visual processing, these studies have predominantly focused on the domain of visual sensitivity. Causal effects of pupil size on visual acuity, therefore, remain poorly understood. By manipulating peripheral background luminance levels and target stimulus contrast while simultaneously recording pupillometry and EEG signals, we examined how absolute pupil size affects visual discrimination and visually evoked potentials (VEP) in a task using optotype mimicking the Snellen eye chart, the most common assessment of visual acuity. Our findings indicate that both higher background luminance levels and higher target contrast were associated with improved target discrimination and faster correct reaction times. Moreover, while higher contrast visual stimuli evoked larger VEPs, the effects of pupil size on VEPs were not significant. Additionally, we did not observe inter-individual correlations between absolute pupil size and discrimination performance or VEP amplitude. Together, our results demonstrate that absolute pupil size, regulated by global luminance level, played a functional role in enhancing visual discrimination performance in an optotype discrimination task. The differential VEP effects of pupil size compared to those of stimulus contrast further suggested distinct neural mechanisms involved in facilitating visual acuity under small pupils.

## Introduction

Pupil size has become a popular index for behavioral and neuroscientific investigations because it is modulated by various sensory, cognitive and affective processes (Strauch et al., 2022; Joshi and Gold, 2020). Notably, the primary function of the pupil is to control the amount of light entering the eye, optimizing the trade-off between visual acuity (the ability to perceive fine details of visual stimuli) and sensitivity (the ability to detect visual stimuli) for effective visual processing (Denton, 1956; Campbell and Gregory, 1960; Woodhouse and Campbell, 1975; Laughlin, 1992). Light enters the eye through the cornea and passes through the pupil before reaching the retina (May et al., 2019; McDougal and Gamlin, 2015; Loewenfeld, 1999), from where relevant visual signals are then transmitted to the visual cortex and subcortical areas to guide behavior. Although this mechanism is fundamental in the field of visual neuroscience, limited research has directly examined the role of pupil size in visual processing.

It is suggested that larger pupil size in dark environments can increase visual sensitivity by allowing more light into the eye, while smaller pupil size in bright environments may enhance visual acuity by reducing optical aberrations (Denton, 1956; Campbell and Gregory, 1960; Woodhouse and Campbell, 1975; Laughlin, 1992; Mathôt, 2020). To test that human pupil light reflex may serve to optimize visual acuity and sensitivity across different luminance levels experimentally, seminal studies have demonstrated that participants detect or discriminate targets most effectively when the size of an artificial aperture closely matches the natural pupil diameter at a given luminance level (Campbell and Gregory, 1960; Woodhouse and Campbell, 1975). Subsequent research manipulating brightness levels to alter pupil size consistently reports better target detection performance associated with larger pupil size (Eberhardt et al., 2022; Wang et al., 2021; Mathôt and Ivanov, 2019). For example, manipulating peripheral background luminance in a visual detection task resulted in higher response accuracy at lower luminance levels (Wang et al., 2021). Additionally, the effects of absolute pupil size on task performance were more pronounced in the generation of visually-guided saccades compared to volitional saccades (Cherng et al., 2021). These results provide empirical evidence supporting the idea that larger pupil size enhance visual sensitivity.

The evidence linking smaller pupil size to enhanced visual acuity is less conclusive (Mathôt and Ivanov, 2019; Ajasse et al., 2018). Some research has found no correlation between absolute pupil size and discrimination performance for a parafoveal target (Ajasse et al., 2018). While a letter discrimination task (discriminating between upper and lower case letters) showed improved performance with smaller pupil size, neither orientation discrimination nor word discrimination was enhanced by smaller pupils (Mathôt and Ivanov, 2019). These findings raise questions about the variability and task-dependence of the relationship between pupil size and visual acuity. Therefore, it is important to examine this relationship using a visual discrimination task similar to those widely used in eye clinics to measure visual acuity.

To understand the causal relationship between pupil size and visual processing, a few studies have concurrently recorded pupil size and electroencephalography (EEG) signals to directly measure visuocortical activity induced by visual stimuli (Bieniek et al., 2013; Bombeke et al., 2016; Suzuki et al., 2019; Mathôt et al., 2023; Thigpen et al., 2018). While these studies have generally found effects of pupil size on visually evoked activity, primarily visually evoked potentials (VEP) (Bieniek et al., 2013; Bombeke et al., 2016; Suzuki et al., 2019; Mathôt et al., 2023), most prior studies have utilized detection-type or task-free paradigms to examine the effects of pupil size on visual sensitivity. The role of pupil size on visually evoked activity during visual discrimination remains largely unexplored. Notably, although the relationship between VEPs and visual acuity is complex and modulated by various factors, research has generally obtained a correlation between higher visual acuity and larger VEP amplitudes (Zheng et al., 2020; Hamilton et al., 2021). This raises an intriguing question: are pupil size effects on VEPs during visual discrimination opposite to those observed during visual detection, as demonstrated behaviorally by Campbell and Gregory (1960)?

**FIGURE 1:**
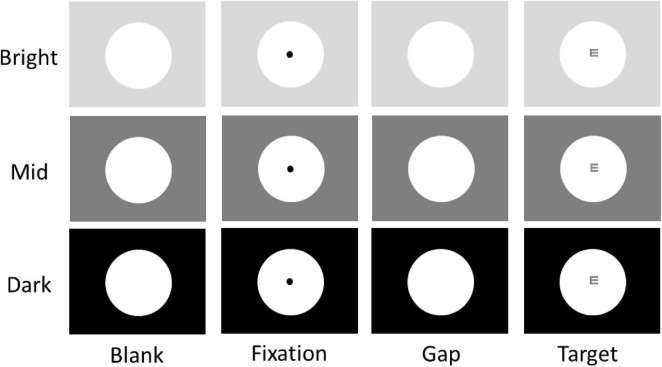
Experimental paradigm. Each trial began with the appearance of a central FP within a large white circle (151 cd/m^2^) on one of three possible background luminance levels (Bright: 137 cd/m^2^; Mid: 36 cd/m^2^; Dark: 0.1 cd/m^2^). After a delay, the central FP was removed for 100 ms (gap) before the optotype target stimulus appeared. Participants were required to report the orientation of the optotype using the four arrow keys (Up, Down, Left, Right) on a keyboard. Note that the optotype shown here is for illustration purposes only.

The goal of the present study is to investigate the role of pupil size on visual processing during visual discrimination through concurrent recording of human pupil size and EEG signals. We use a task involving optotypes (Figure 1) to examine visual discrimination because this type of eye chart is widely used in eye clinics, making it an effective index for representing visual discrimination performance (Intoy and Rucci, 2020). Peripheral background luminance levels were manipulated to alter pupil size (Eberhardt et al., 2022; Wang et al., 2021; Mathôt and Ivanov, 2019), and moreover, target visual contrast was also manipulated (Zhang and Luo, 2012; Schadow et al., 2007; Vassilev et al., 1994; Muller et al., 1988; Bodis-Wollner et al., 1972; Andersen et al., 2012) so that we could compare the effects of both pupil size and target contrast on task performance and VEPs. We hypothesize that if smaller pupil size results in better retinal image quality during visual discrimination, smaller pupil size should correlate with better task performance and larger VEPs. Specifically, we anticipate observing similar effects on discrimination performance and VEPs between pupil size and stimulus contrast.

## Materials and methods

### Experimental setup

All experimental procedures were reviewed and approved by the Institutional Review Board of the Taipei Medical University, Taiwan, and were in accordance with the Declaration of Helsinki (World Medical Association, 2001). 50 participants (mean age = 22.7 years; SD = 2.8 years; 26 females) were recruited via an advertisement posted on the university website. This sample size was chosen based on previous studies with comparable pupil size and EEG measurements and trial numbers per participant (Causse et al., 2016; Lauffs et al., 2020; de Gee et al., 2021). Participants had normal or corrected-to-normal vision and were naïve regarding the purpose of the experiment. Participants provided informed consent and were compensated financially for their participation.

### Recording and apparatus

During testing, participants were seated in a dark room, with their head stabilized in a chin and forehead rest, and the only light source was the stimulus display (LCD screen). Eye position and pupil size were measured with a video-based eye tracker (Eyelink 1000 Plus, SR Research, Osgoode, ON, Canada) at a sampling rate of 1000 Hz. Stimuli were viewed monocularly with the left eye while the right eye was covered with a patch, mimicking the standard practice of Snellen eye chart test. Following our previous study (Chang et al., 2023), we used the 32-channel Brain Products system (actiCHamp, Brain Products) to record EEG signals at a sampling rate of 1000 Hz. Electrodes were positioned based on the standard international 10-20 system, with a common vertex reference placed between Cz and CPz. Additionally, a ground electrode was positioned anterior to Fz on the forehead. Electrode impedances were maintained below 5 KΩ. Autonomic responses were also recorded by BIOPAC MP36 (BIOPAC systems, USA), but these were not included in the current study. The presentation of stimuli and acquisition of data were controlled by Eyelink Experiment Builder and Eyelink software. Experiment Builder sent TTL pulses to mark experiment events on the EEG and BIOPAC systems, ensuring accurate alignment of trial stimulus events in both EEG and BIOPAC recordings. Stimuli were displayed on an LCD monitor with a screen resolution of 1920 x 1080 pixels and a refresh rate of 60 Hz, resulting in a viewing angle of 37° x 21°. The distance from the eyes to the monitor was maintained at 80 cm.

### Visual acuity task

We used a task (Figure 1), mimicking the Snellen eye chart test, to examine visual acuity performance. Each trial began with the appearance of a large white circle (10° diameter, 151 cd/m^2^) in the center of the screen with 3 possible peripheral background luminance levels (bright: 137 cd/m^2^; mid: 36 cd/m^2^; dark: 0.1 cd/m^2^) for 1.6 – 1.8 s. Then, a black fixation point (FP) (0.1° diameter, 0.01 cd/m^2^) appeared in the center of the large white circle. After 1 – 1.2 s of central fixation, the FP disappeared for 100 ms (gap) before the target stimulus (an optotype, 0.104°) appeared in the center of the screen for 1500 ms. The gap period between FP disappearance and optotype (referred to as target) appearance was inserted so that the size of the pupil could only influence visual signals induced by the target stimulus. Tumbling-E optotypes were used; each optotype was oriented in one of four possible directions (up, down, left, or right) at one of two luminance levels (37 or 138 cd/m^2^, referred to as high or low contrast, respectively). Participants were asked to report the orientation of the optotype using the four arrow keys (Up, Down, Left, Right) on a keyboard as quickly and accurately as possible. Building upon previous research that manipulated peripheral luminance levels to alter pupil size (Wang et al., 2021; Mathôt and Ivanov, 2019), the visual contrast of the target at the high (or low) contrast condition remained consistent across three luminance levels. Participants performed several practice trials to familiarize themselves with the task and its requirements (e.g., maintain central fixation) before starting the experiment. The experiment consisted of 360 trials. Target contrast condition (high and low), peripheral background luminance condition (bright, mid, and dark), and target orientation condition (up, down, left, and right) were randomly interleaved with equal frequency. Trials with different optotype orientations were combined for data analysis.

### Data analysis

Reaction time (RT) was defined as the time from optotype appearance to the onset of the key press. Trials were scored as correct if the response arrow key matched the optotype orientation. Participants were required to maintain central fixation during each trial. Trials with saccades (eye velocity exceeding 30°/s) during the period from 100 ms before target appearance to the onset of the key press were removed, resulting in the exclusion of 0.5% of trials. Notably, although the orientation of microsaccades can affect visual acuity performance (Intoy and Rucci, 2020; Rucci and Victor, 2015; Rucci and Poletti, 2015), we did not exclude trials with microsaccades for several reasons. First, including more trials increases statistical power for data analysis. Second, unlike previous research that used a Dual Purkinje Image eye-tracker with higher accuracy in measuring eye position, we used a video-based eye-tracker, which is less accurate in detecting microsaccade orientation. Finally, this aspect was beyond the focus of our study. Four participants were excluded because of missing more than 50% of the trials due to technical issues, yielding 46 participants for data analysis. To maintain an accurate measure of pupil size, participants were required to maintain central fixation during the task. A well-established method was used for pupil data preprocessing (Kret and Sjak-Shie, 2019). Specifically, invalid data time points (e.g., blinks) were identified, and pre- and post-invalid pupil values were used to perform a linear interpolation to replace invalid pupil values. Following this, the data were smoothed using a zero-phase low-pass filter with a cut-off frequency of 5 Hz. To investigate the effects of pupil responses on visual acuity performance, absolute pupil size (referred to as absolute pupil diameter) was calculated as the mean pupil size during an epoch from 50 ms before to 50 ms after target (optotype) appearance (referred to as the target epoch).

Following our previous study for EEG offline-preprocessing (Chang et al., 2023), we conducted EEG data analysis using the Automagic pipeline (Pedroni et al., 2019) in MATLAB. In accordance of established procedures (Bigdely-Shamlo et al., 2020a; Bigdely-Shamlo et al., 2020b), PREP was utilized to identify and correct for bad electrodes, followed by detrending the data with a 1 Hz high-pass filter. Subsequently, filtering to remove 60 Hz line noise and its harmonics was performed, and the data was re-referenced using the robust averaging referencing method. Spherical interpolation was applied to replace any detected bad channels. The EEG data underwent bandpass filtering within the 0.1–30 Hz range. To address artifacts stemming from eye blinks, heartbeat, and muscle motion, independent component analysis was employed, utilizing the Multiple Artifact Rejection Algorithm (Delorme and Makeig, 2004; Winkler et al., 2011). Subsequently, EEG signals were segmented into epochs aligned with stimulus onset (time on target onset), spanning 3 seconds, including a 1000 ms pre-target interval and a 2000 ms post-stimulus interval. Baseline correction to the pre-stimulus interval (pre-stimulus period of 200 ms) was conducted. Epochs exhibiting activities exceeding ± 150 μV were excluded from subsequent analysis. After epoch rejection, no participants had a percentage of valid epochs below 99%. Note that trials with RT values beyond 1.5 times the interquartile range (the difference between upper and lower quartiles) below the lower quartile or above the upper quartile were excluded from analysis. The above criteria together resulted in the removal of 6.4% of trials.

Visually-evoked event related potential (ERP) components were specifically used to examine the relationship between pupil size and visually evoked activity, because VEPs are modulated by stimulus contrast (Zhang and Luo, 2012; Schadow et al., 2007; Vassilev et al., 1994; Muller et al., 1988; Bodis-Wollner et al., 1972; Andersen et al., 2012), as one of the most primitive components of saliency (Itti and Koch, 2001; Itti et al., 1998; Borji et al., 2013), allowing us to compare the effects of pupil size with those of stimulus contrast. Similar to previous research (Rygvold et al., 2021; Rygvold et al., 2022), we focused on three components: C1, P1, and N1, and these VEPs were measured using data of electrodes in the occipital scalp, including the O1, Oz, and O2. Following the literature, three time windows were selected to capture the peak for each component (C1: 90–110 ms, P1:130–160 ms, N1: 180–205 ms). Latencies and topographical distributions of different VEP components were qualitatively similar to those observed in previous research (Rygvold et al., 2021; Rygvold et al., 2022).

A two-way repeated-measure ANOVA was used to examine the effects of background luminance condition (bright, mid, dark) and target (optotype) contrast (high and low) on the accuracy and speed of manual responses as well as on VEP amplitudes. Effect sizes (partial eta squared) are reported where appropriate. Inter-individual correlational analyses were used to examine the inter-individual relationship between absolute pupil diameter and visual discrimination performance or VEP responses. Statistical tests were performed using the lmer function in R Project (R Core Team, 2020; Rstudio Team, 2019), JASP Team (2019), and MATLAB (The MathWorks Inc., Natick, MA, USA).

## Results

### Effects of peripheral background luminance level on absolute pupil size

**FIGURE 2:**
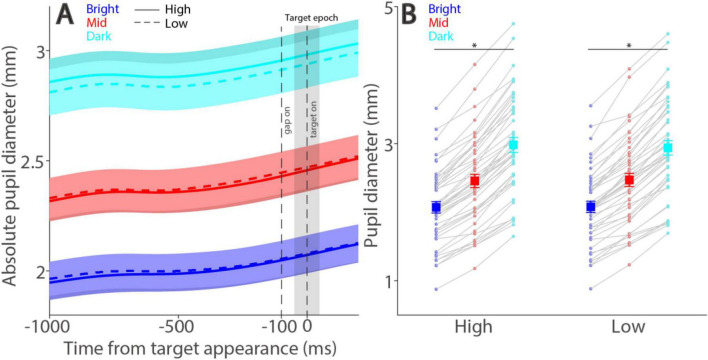
Effect of background luminance on absolute pupil size. Absolute pupil diameter following optotype (target) appearance for each background luminance level **(A)**. Mean absolute pupil diameter (−50 to 50 ms after optotype onset) for each background luminance level and contrast condition **(B)**. In A, the shaded colored regions surrounding the pupillary response curves represent the ± standard error range (across participants). The target epoch is shaded in gray. In B, the large squares and error bars represent the mean values ± standard error across participants. The small dots represent the mean value for each participant. Bright: high background luminance, Mid: middle background luminance, Dark: low background luminance. High: high target visual contrast, Low: low target visual contrast. * indicates statistically significant.

As illustrated in Figure 1, tumbling-E optotypes were used to examine visual discrimination performance, and the size of absolute pupil diameter was manipulated by varying peripheral background luminance levels (see Methods). Pupil size is regulated primarily by the global luminance level, with smaller pupil size observed in higher levels of global luminance (McDougal and Gamlin, 2015; Loewenfeld, 1999). Consistently, here we found that absolute pupil diameter changed as a function of peripheral background luminance level, with smaller pupil diameter in trials with higher levels of peripheral background luminance (Figure 2A). Absolute pupil diameter at the target epoch (50 ms before to 50 ms after optotype appearance) was significantly influenced by background luminance level (Figure 2B, luminance main effect: F(2,90) = 519.610, *p* < 0.001, η_p_^2^ = 0.920). For high contrast stimuli, mean pupil diameters were 2.07, 2.46, and 2.98 mm for the bright, mid, and dark conditions, respectively. For low contrast stimuli, mean pupil diameters were 2.08, 2.47, and 2.94 mm for the bright, mid, and dark conditions, respectively. Because the target contrast condition should not affect absolute pupil diameter before target appearance, any effects related to target contrast are not considered for further analyses. Absolute pupil diameter was not significantly influenced by target contrast (F(1,45) = 1.284, *p* = 0.263, η_p_^2^ = 0.028).

### Effects of peripheral background luminance level and target contrast on performance accuracy and reaction time

**FIGURE 3:**
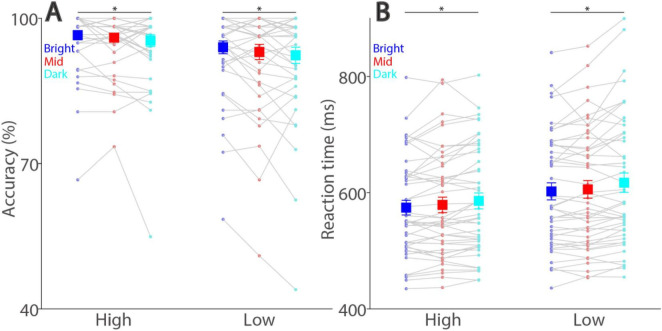
Effect of background luminance and target contrast on task accuracy and reaction time. Discrimination accuracy **(A)** and reaction time **(B)** shown for different target contrast conditions and background luminance levels. The large squares and error bars represent the mean values ± standard error across participants. The small dots represent the mean value for each participant. Bright: high background luminance, Mid: middle background luminance, Dark: low background luminance, Low: low target visual contrast. * indicates statistically significant.

If smaller pupil size increases visual acuity (Laughlin, 1992), then better task performance should be observed with a brighter background (i.e., smaller pupil diameter). As illustrated in Figure 3A, background luminance level significantly affected response accuracy (luminance main effect: F(2,90) = 5.443, *p* = 0.013, η_p_^2^ = 0.108), with lower error rates in trials with higher background luminance levels. For high contrast stimuli, mean accuracies were 97, 96, and 95% with high contrast stimuli, 94, 93, and 92% with low contrast stimuli in the bright, mid, and dark conditions, respectively. Note that these effects of background luminance levels were in the opposite direction of those observed in the visual detection task (Wang et al., 2021), as we previously found lower accuracy in higher background luminance levels. Moreover, background luminance consistently modulated performance accuracy in the two target contrast conditions (simple main effects: all ps < 0.05). In addition to the background luminance modulation, as expected, visual contrast also affected response accuracy with lower error rates in the high contrast condition, compared to the low contrast condition (contrast main effect: F(1,45) = 21.179, *p* < 0.001, η_p_^2^ = 0.320). The interaction was not significant (F(2,90) = 0.336, *p* = 0.709, η_p_^2^ = 0.007).

Peripheral background luminance level also significantly modulated reaction times (RT) (Figure 3B, luminance main effect: F(2,90) = 17.628, *p* < 0.001, η_p_^2^ = 0.281), with mean RTs in correct trials being 574, 579, and 586 ms with high contrast stimuli, being 602, 606, and 617 ms with low contrast stimuli in the bright, mid, and dark conditions, respectively. Again, these background luminance effects were reliably pronounced across two target contrast conditions (simple main effects: all ps < 0.001). Furthermore, visual contrast significantly affected RTs (contrast main effect: F(1,45) = 82.327, *p* < 0.001, η_p_^2^ = 0.647), with faster SRTs for higher contrast stimuli, and interaction between background luminance level and target contras was not significant (F(2,90) = 0.513, *p* = 0.590, η_p_^2^ = 0.011). Together, these results suggested that, in addition to the target contrast effect, differences in absolute pupil diameter systematically modulated visual discrimination performances in the optotype discrimination task, with better task performances with higher luminance or higher target contrast levels.

### Effects of peripheral background luminance level and target contrast on visually-evoked potentials

**FIGURE 4:**
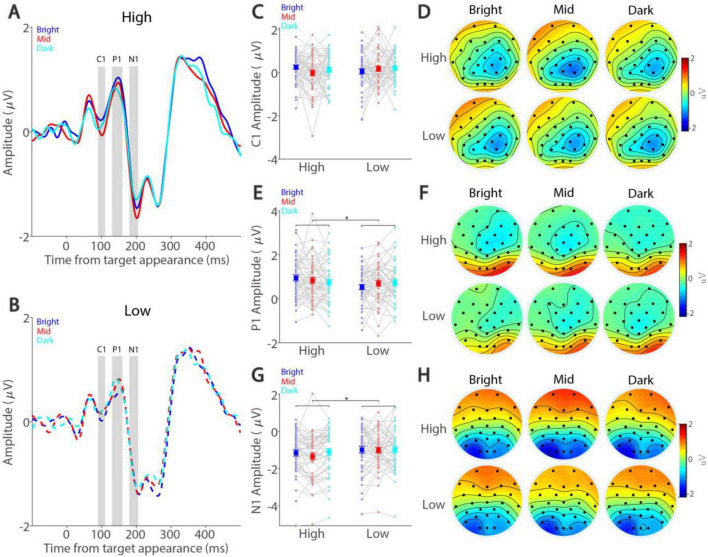
Effect of background luminance and target contrast on visually evoked potentials. Amplitude dynamics for high-contrast **(A)** and low-contrast **(B)** conditions shown for different background luminance levels. Mean amplitude in C1 **(C)**, P1 **(E)**, and N1 **(G)** components shown for different target contrast conditions and background luminance levels. Topographies of mean C1 **(D)**, P1 **(F)**, and N1 **(H)** amplitude shown for different target contrast conditions and background luminance levels. In **(A)** and **(B)**, the gray area represents the epoch selected for analyses. In **(C,E,G)** the large squares and error bars represent the mean values ± standard error across participants. The small dots represent the mean value for each participant. Bright: high background luminance, Mid: middle background luminance, Dark: low background luminance. High: high target visual contrast, Low: low target visual contrast. * indicates statistically significant.

We next examined whether smaller pupil size was associated with higher VEPs during task performance. Figures 4A, B illustrate dynamics of ERP signals averaged across three occipital electrodes (see Methods) for the high and low target contrast conditions, respectively. As shown in Figure 4C, the amplitude of C1 peaked at around 96 ms and was not modulated by either peripheral background luminance level (F(2,90) = 0.411, *p* = 0.650, η_p_^2^ = 0.009) or target contrast (F(1,45) = 0.136, *p* = 0.714, η_p_^2^ = 0.003). The interaction between background luminance level and target contrast was also not significant (F(2,90) = 1.646, *p* = 0.199, η_p_^2^ = 0.035). Moreover, topographical distribution for C1 across the background luminance levels was observed in parietal and occipital electrodes (Figure 4D). Figure 4E shows the amplitude of P1 with a positive deflection peaking at around 147 ms, with higher target contrast correlating with higher P1 (F(1,45) = 6.141, *p* = 0.017, η_p_^2^ = 0.120). In contrast, peripheral background luminance level did not significantly affect P1 (F(2,90) = 0.050, *p* = 0.949, η_p_^2^ = 0.001). The interaction was not significant (F(2,90) = 2.733, *p* = 0.075, η_p_^2^ = 0.057). Topographical distribution for P1 was mainly observed at occipital electrodes (Figure 4F). Similarly, target contrast significantly modulated the N1 component (Figure 4G), peaking at around 203 ms, with higher target contrast correlating with higher amplitudes (F(1,45) = 9.701, *p* = 0.003, η_p_^2^ = 0.177). However, peripheral background luminance level did not significantly affect N1 amplitude (F(2,90) = 1.289, *p* = 0.281, η_p_^2^ = 0.028), and the interaction was also not significant (F(2,90) = 0.978, *p* = 0.378, η_p_^2^ = 0.021). Topographical distribution for N1 was mainly observed at occipital electrodes (Figure 4H). Together, these results suggested that target contrast, but not absolute pupil size, significantly affected VEPs induced by target stimuli.

We further conducted inter-individual correlational analyses to examine whether absolute pupil size correlates with visual discrimination performance or VEPs at the inter-individual level. As shown in Supplementary Figures 1, 2, there were no inter-individual correlations between absolute pupil diameter and visual discrimination performance or VEPs.

## Discussion

The present study investigated the influence of absolute pupil diameter, modulated by peripheral background luminance levels, on visual discrimination performance and visually evoked potentials (VEPs) in an optotype discrimination task. We hypothesized that in visual discrimination, a smaller pupil size should result in better task performance and larger VEPs. Here, we observed a clear modulation of absolute pupil diameter by background luminance levels, with larger pupil diameters in trials with lower background luminance levels. As expected, target contrast significantly affected discrimination performance, with higher accuracy and faster RTs in the high contrast condition. More importantly, differences in pupil diameter induced by background luminance levels also systematically affected visual discrimination performance. Target discrimination rates were higher, and correct RTs were faster under higher background luminance conditions. Notably, while target contrast significantly modulated P1 and N1 amplitudes, with higher amplitudes obtained in the high contrast condition, peripheral background luminance levels did not significantly affect these VEPs. Moreover, absolute pupil size did not exhibit inter-individual correlations with discrimination performance or VEPs. In summary, our findings demonstrate the role of absolute pupil size on visual discrimination performance, but not on VEPs, in an optotype discrimination task. This implicates the functional role of absolute pupil size, regulated by global luminance levels, in enhancing visual acuity for foveal visual processing. However, the facilitative effects of pupil size were not simply mediated by inducing larger VEPs as those of stimulus contrast.

### Linking absolute pupil size to visual discrimination

Consistent with previous findings from the letter discrimination task (Mathôt and Ivanov, 2019), we found that smaller absolute pupil size (i.e., higher peripheral brightness levels) was associated with better discrimination performance, as measured by response accuracy and reaction times. These results demonstrate a correlation between smaller pupil size and improved visual discrimination in a task similar to those commonly used in clinics to measure visual acuity. Additionally, we explored whether absolute pupil size correlates with task performance at the inter-individual level and found no such correlations. This finding suggests that individuals with smaller pupil size do not necessarily perform better in visual discrimination compared to those with larger pupil size. Note that there were individual differences in visual acuity among participants, with those having better visual acuity generally showing higher accuracy and faster reaction times, though these effects were not statistically significant (Supplementary Figure 3). This variability in acuity could influence the inter-individual correlational results, and future studies with better control of visual acuity are necessary to further address this question.

Notably, a body of research has shown intriguing center-surround interactions among stimulus size, spatial frequency, and contrast (Angelucci and Bressloff, 2006; Tadin, 2015; Sceniak et al., 1999; Cavanaugh et al., 2002). For example, while larger stimuli typically enhance visual perception, increasing the size of a high-contrast stimulus, as opposed to a low-contrast stimulus, in a motion detection task actually raises the threshold for detecting motion direction (Tadin et al., 2003). These counterintuitive results can be well explained by center-surround antagonism: when a large high-contrast stimulus falls into both the center and surround regions of neurons, inhibition from the surround counteracts excitation from the center, leading to reduced overall neuronal activity and poorer motion perception. Our study, by manipulating background luminance and target contrast, possibly engaged these center-surround mechanisms. If absolute pupil size could alter visual responses in a similar manner to that of visual contrast, one might expect to observe similar center-surround interactions resulting from changes in absolute pupil size. However, we found that the influence of pupil size on VEPs differed from the effects of stimulus contrast, suggesting the complexity of these interactions involving pupil size. Nevertheless, further research is indeed needed to disentangle the contributions of absolute pupil size as well as other visual attributes on center-surround mechanisms to better understand their role in supporting visual perception.

### Relationship between pupil size and visually evoked potentials

To directly investigate the functional role of pupil size on visual processing in humans, a limited number of studies have concurrently recorded pupillometry and EEG signals (Bieniek et al., 2013; Bombeke et al., 2016; Suzuki et al., 2019; Mathôt et al., 2023; Thigpen et al., 2018). In EEG, VEPs have been regularly used to measure visuocortical activity induced by visual stimuli (Creel, 2019; Sokol, 1976; Tobimatsu and Celesia, 2006). Stimulus contrast, as one of the most primitive components of saliency (Itti and Koch, 2001; Itti et al., 1998; Borji et al., 2013), significantly modulates VEPs, with higher contrast correlating with higher VEP amplitudes (Zhang and Luo, 2012; Schadow et al., 2007; Vassilev et al., 1994; Muller et al., 1988; Bodis-Wollner et al., 1972; Andersen et al., 2012). Consistently, in our study, we obtained larger P1 and N1 amplitudes in the high contrast condition compared to the low contrast condition. While the link between visual acuity and VEPs is complicated and VEPs are limited in the spatial resolution, compared to other techniques (e.g., single unit, LFP, fMRI), to indicate the locus of neural correlates, a positive relationship between visual acuity and VEP responses has often observed (Zheng et al., 2020; Hamilton et al., 2021). Contrary to our prediction, VEP amplitudes were not significantly modulated by peripheral luminance levels, even though absolute pupil size were dramatically different in different luminance conditions. These results differ from previous studies showing an effect of pupil size on visuocortical activity (Bieniek et al., 2013; Bombeke et al., 2016; Suzuki et al., 2019; Mathôt et al., 2023). We think that these differences could be partly due to the requirement of different task demands. Previous studies have mostly utilized detection-type or task-free paradigms, but in the current study, participants were required to perform a visual discrimination task. Pupil size effects on VEPs could be more pronounced in the visual detection task. It is also important to note that effects of pupil size on visuocortical activity are highly variable. For example, lower steady-state VEP amplitudes have been obtained with smaller pupils in one study (Suzuki et al., 2019), whereas larger C1 amplitudes have been obtained with smaller pupils in another study (Bombeke et al., 2016). A study examining pupil size effects on steady-state VEPs under constant luminance has found no effects of pupil size on steady-state VEPs (Thigpen et al., 2018). Notably, in these studies, visuocortical activity measured through EEG has been evoked by visual stimuli with radically different conditions under different task requirements. For example, visual stimuli have been presented in the fovea in some studies but presented in the parafovea or periphery in other studies, and visual stimuli were task-relevant in some studies but were irrelevant in others. Taken together, these findings suggest that caution should be exercised when interpreting the role of absolute pupil size in visual processing, and research would benefit from using more consistent experimental paradigms/methods.

### Limitations and future directions

The current paradigm allowed us to investigate the impact of absolute pupil size on visual discrimination performance using optotype stimuli. To streamline the number of experimental conditions to achieve greater statistical power for analysis, our study was limited to using only the left eye and a fixed size of optotype stimuli. Future research is warranted to explore the other eye and different sizes of optotype stimuli. Moreover, research has demonstrated that microsaccades are involved in visual discrimination performance, and fixational eye movements are not identical across neurotypical individuals under monocular viewing conditions (Intoy and Rucci, 2020; Rucci and Victor, 2015; Rucci and Poletti, 2015). While fixational eye movements are beyond the scope of the current study, future research is certainly needed to address this question. Additionally, eye dominance was not controlled for across participants. Although eye dominance did not significantly affect accuracy or reaction times (Supplementary Figure 4), this factor likely plays a role in visual discrimination performance and warrants further investigation. Additionally, our study employed only three peripheral background luminance conditions, and the range of absolute pupil size primarily fell within the 2 to 3 mm diameter range. It is essential to expand the exploration of absolute pupil size over a broader range to ensure a comprehensive understanding of its effects on visual acuity. Furthermore, the current study is constrained by its relatively small cohort, consisting solely of young adults. Future research should involve larger study cohorts spanning a wider age range to provide more generalizable findings. Additionally, given the limitations of VEP spatial resolution, it is essential to explore this topic using techniques such as neuronal recordings and fMRI to better understand the role of pupil size, regulated by background luminance levels, on visual activity in visual-related areas within the context of visual acuity. In summary, while our findings support the hypothesis that smaller pupil size may enhance visual discrimination performance in an optotype discrimination task, it is crucial to recognize differences between modulations of absolute pupil size and stimulus contrast on VEPs. Future investigations using other means to measure visuocortical activity, such as neuronal recordings in behaving animals, are necessary to fully understand how absolute pupil size modulates visual responses and subsequently affects visual discrimination performance.

## Data Availability

The raw data supporting the conclusions of this article will be made available by the authors, without undue reservation.
