# Supplementary material for: Investigating causal effects of pupil size on visual discrimination and visually evoked potentials in an optotype discrimination task
Source: Front Neurosci. 2024 Oct 1;18:1412527. doi: 10.3389/fnins.2024.1412527 (PMC11473405; doi:10.3389/fnins.2024.1412527)
Supplement: Supplementary file 1 [file Data_Sheet_1.pdf]

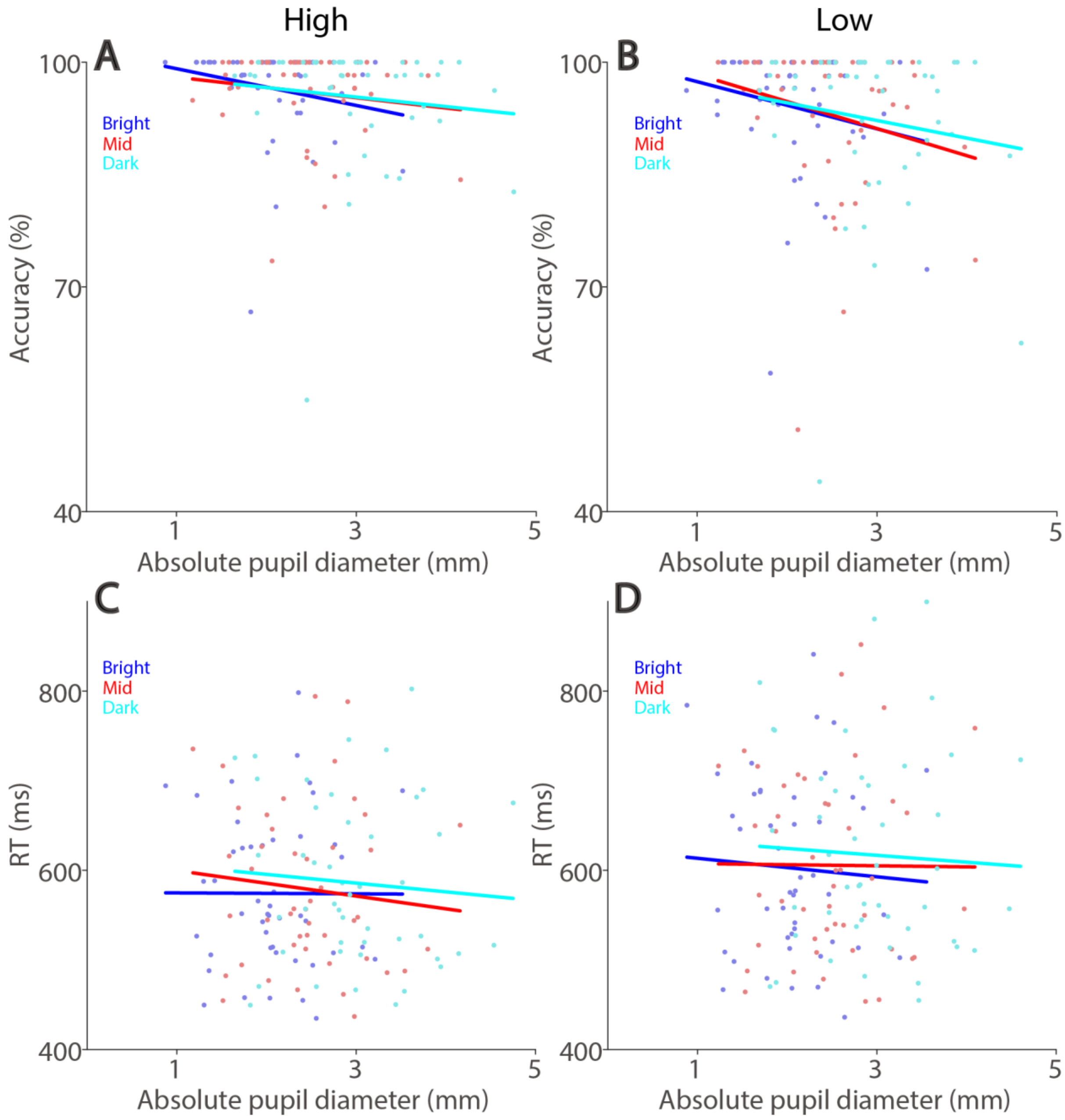

Supplementary Figure 1. Inter-individual correlation between absolute pupil size and task performance. Inter-individual correlations between absolute pupil size and accuracy for each background luminance level for high contrast **(A)** and low contrast **(B)** conditions, and between absolute pupil size and reaction time for each background luminance level for high contrast **(C)** and low contrast **(D)**. Colored lines indicate regression lines. The small dots represent the mean value for each participant. Bright: high background luminance, Mid: middle background luminance, Dark: low background luminance. High: high target visual contrast, Low: low target visual contrast.

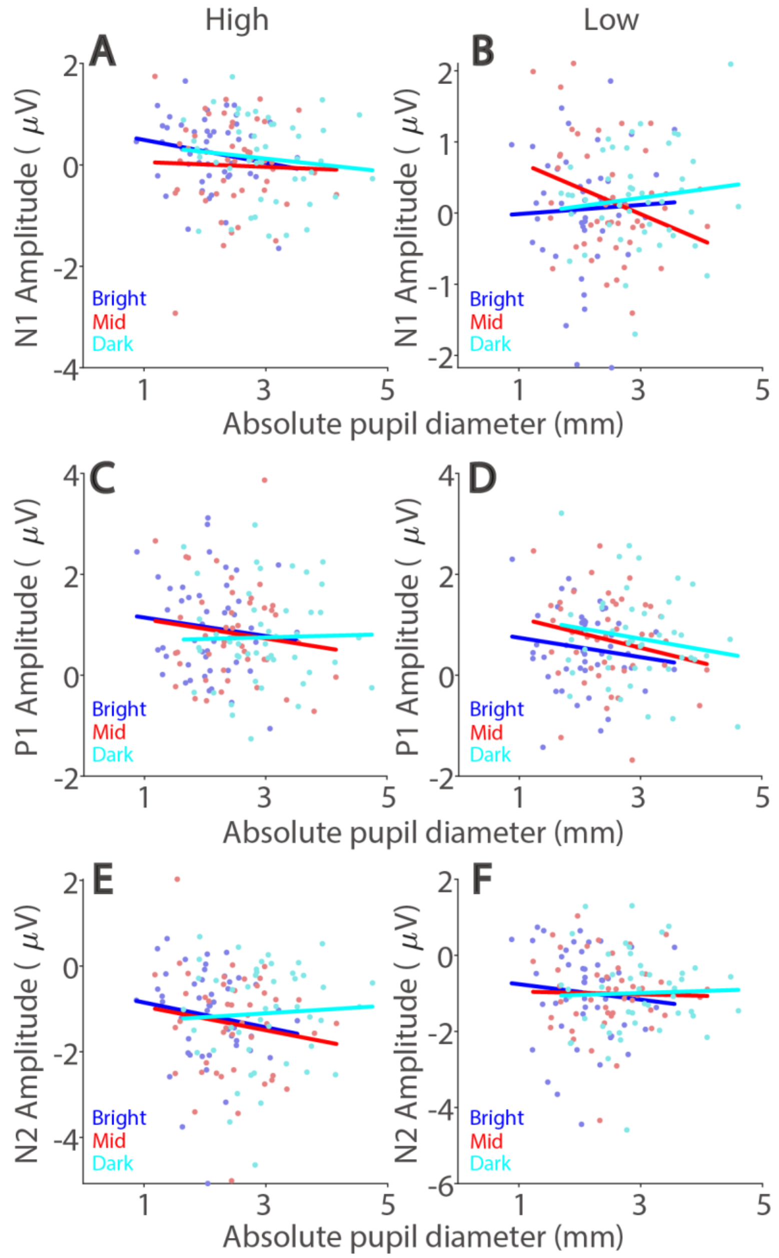

Supplementary Figure 2. Inter-individual correlation between absolute pupil size and VEP. Inter-individual correlations between absolute pupil size and C1 amplitude for each background luminance level for high contrast **(A)** and low contrast **(B)** conditions, between absolute pupil size and P1 amplitude for each background luminance level for high contrast **(C)** and low contrast **(D)**, and between absolute pupil size and N1 amplitude for each background luminance level for high contrast **(E)** and low contrast **(F)**. Colored lines indicate regression lines. The small dots represent the mean value for each participant. Bright: high background luminance, Mid:

middle background luminance, Dark: low background luminance. High: high target visual contrast, Low: low target visual contrast.

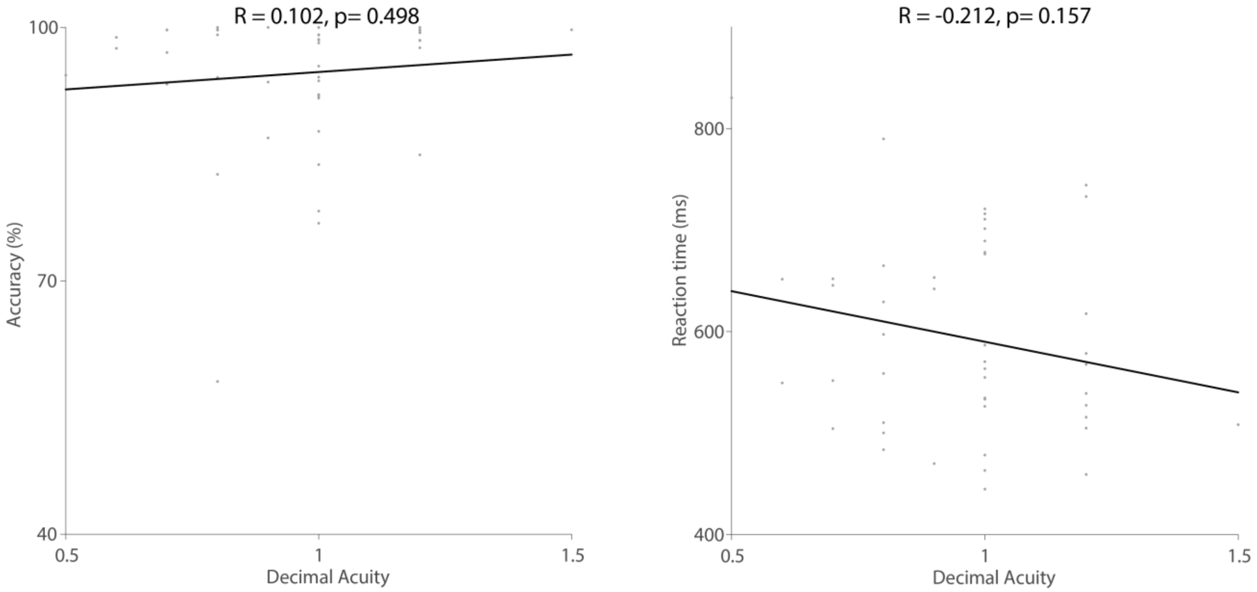

Supplementary Figure 3. Inter-individual correlation between visual acuity and task performance. Inter-individual correlations between visual acuity and accuracy and reaction time (all conditions were collapsed). Black lines indicate regression lines. The small dots represent the mean value for each participant. Note that in Taiwan, visual acuity is measured using the decimal system, where 1.0 corresponds to normal vision. To translate these values to the Snellen fraction (e.g., 20/20), e.g., 1.0 decimal = 20/20 Snellen; 0.8 decimal = 20/25 Snellen; 0.6 decimal = 20/33 Snellen.

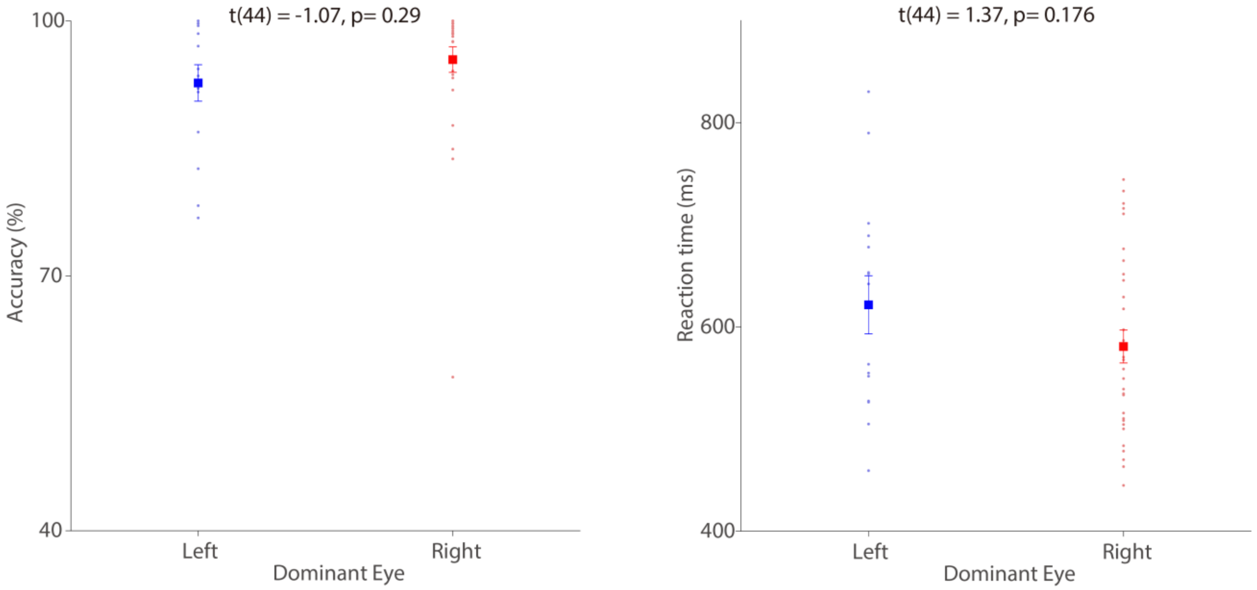

Supplementary Figure 4. Eye dominance effects on accuracy and reaction time. Differences between participants with left eye dominance (N = 15) and those with right eye dominance (N = 31) on accuracy and reaction time (all conditions were collapsed). The large squares and error bars represent the mean values ± standard error across participants. The small dots represent the mean value for each participant.
